# Progranulin antibodies entertain a proinflammatory environment in a subgroup of patients with psoriatic arthritis

**DOI:** 10.1186/ar4406

**Published:** 2013-12-10

**Authors:** Lorenz Thurner, Marina Zaks, Klaus-Dieter Preuss, Natalie Fadle, Evi Regitz, Mei Fang Ong, Michael Pfreundschuh, Gunter Assmann

**Affiliations:** 1 Department of Internal Medicine I, José Carreras-Center for Immuno- and Gene Therapy, University of Saarland Medical School, Kirrbergerstr., D-66421 Homburg, Saar, Germany; 2 Institute of Medical Biostatistics, Saarland University, Kirrbergerstr., D-66421 Homburg, Saar, Germany

## Abstract

**Introduction:**

Psoriatic arthritis (PsA) is a distinctive inflammatory arthritis which may typically develop in a subgroup of individuals suffering from psoriasis. We recently described progranulin autoantibodies (PGRN-Abs) in the sera of patients with different autoimmune diseases including seronegative polyarthritis. In the present study we investigated the occurrence of PGRN-Abs in PsA.

**Methods:**

PGRN-Abs were determined in 260 patients with PsA, 100 patients with psoriasis without arthritic manifestations (PsC) and 97 healthy controls using a recently described ELISA. PGRN plasma levels were determined from subgroups by a commercially available ELISA-kit. Possible functional effects of PGRN-antibodies were analysed *in vitro* by tumour necrosis factor (TNF)-α mediated cytotoxicity assays using WEHI-S and HT1080 cells.

**Results:**

PGRN-Abs were detected with relevant titres in 50/260 (19.23%) patients with PsA, but in 0/100 patients with psoriasis without arthritic manifestations (*P* = 0.0001). All PGRN-Abs belonged to immunoglobulin G (IgG). PGRN-Abs were significantly more frequent in PsA patients with enthesitis or dactylitis. PGRN-Abs were also more frequent in PsA patients receiving treatment with TNF-α-blockers than in patients treated without TNF-α-blockers (20.8% versus 17.4%; *P* = 0.016). PGRN plasma levels were significantly lower in PGRN-Ab-positive patients with PsA than in healthy controls and patients with psoriasis without arthritic manifestations (*P* < 0.001), indicating a neutralizing effect of PGRN-Abs. Moreover cytotoxicity assays comparing PGRN-antibody positive with negative sera from matched patients with PsA, clearly showed a proinflammatory effect of PGRN antibodies.

**Conclusion:**

Neutralizing PGRN-Abs occur with relevant titres in a subgroup of patients with PsA, but not in patients without arthritic manifestations (PsC). PGRN-Ab-positive patients had more frequent enthesitis or dactylitis. TNF-α-induced cytotoxicity assays demonstrated that the protective effects of progranulin were inhibited by serum containing PGRN-Abs. This suggests that PGRN-Ab might not only be useful as a diagnostic and prognostic marker, but may provide a proinflammatory environment in a subgroup of patients with PsA.

## Introduction

Psoriatic arthritis (PsA) is a distinctive inflammatory form of arthritis that may develop in 20% to 25% of individuals with psoriasis [1]. In addition to manifestations of psoriasis in the skin, patients with PsA may present with mild to very severe development of oligoarthritis and/or polyarthritis, enthesitis, dactylitis or axial skeletal manifestations similar to spondyloarthritis. PsA has been considered a seronegative inflammatory arthritis according to the diagnostic criteria first published by Moll *et al*. in 1973 [2] and then redefined by the Classification *Criteria* for Psoriatic Arthritis (CASPAR) [3]. All definitions of PsA have in common the seronegative status of the disease because autoantibodies (Abs) such as rheumatoid factor (RF), anticyclic citrullinated autoantibodies and antinuclear autoantibodies are usually absent in PsA. Hence, in contrast to rheumatoid arthritis, autoreactive B lymphocytes are believed to play only a minor role in PsA [4]. Regarding the occurrence of autoantibodies in PsA, increased frequencies of thyreoglobulin Abs (14.29%) and thyroid peroxidase Abs (23%) were reported in PsA, which was explained by a relatively high comorbidity rate, with 26% of patients with PsA having autoimmune thyroiditis [5]. In another study, 20S proteasome autoantibodies were more frequently detected in PsA patients (27.8%) than in in healthy controls (0%), as well as more frequently in systemic lupus erythematosus patients (42%) than in rheumatoid arthritis patients (5%) [6]. However, the numbers of patients were small in these studies (36 PsA patients and 30 healthy controls) [6], and, in both studies, no patients with psoriasis without arthritic manifestations (PsC) were included. To date, no specific serological markers discriminating patients with PsA from patients with PsC have been identified. Nevertheless, a small but significant occurrence of B lymphocytes was reported in the skin of patients with PsA, but not in patients with PsC [7].

Recently, we discovered progranulin autoantibodies (PGRN Abs) in a protein array-based screening of plasma from various primary vasculitides and found evidence that these PGRN Abs have a neutralizing effect on PGRN plasma levels [8]. PGRN is a secreted precursor protein that is cleaved at the linker regions between individual granulins by neutrophil elastase [9], proteinase 3 [10], matrix metalloproteinase 12 [11], matrix metalloproteinase 14 [9] and ADAMTS-7 (a disintegrin and metalloprotease with thrombospondin motif 7) [12]. Until recently, most research on PGRN had focused on its role in neurodegenerative diseases such as frontotemporal lobe dementia [13]. However, since Tang *et al*. [14] showed that PGRN is a high-affinity ligand of the tumour necrosis factor α (TNF-α) receptors 1 and 2 (TNFR1 and TNFR2) and that its anti-inflammatory effect is caused by direct inhibition of these receptors, PGRN has increasingly become the focus of research on autoimmune diseases.

Recently, Chen *et al*. [15] challenged the notion of this interaction of PGRN with TNFR1 and TNFR2 previously reported by Tang *et al*. [14], as they could not reproduce the interaction of PGRN with TNFR1 and TNFR2. However, they did not question the anti-inflammatory effect of PGRN [10]. Tang *et al*. responded in a letter to the editor that Chen *et al*. utilized PGRN, which might be folded improperly. Furthermore, Tang *et al*. stated that validation of recombinant PGRN’s functionality based only on its C-terminal binding to sortilin would be insufficient to determine its quality regarding its other biological functions, which are not primarily mediated by PGRN’s C-Terminus. Subsequently, Jian *et al*. showed in detail that PGRN binds as TNF-α to cysteine-rich domain 2 (CRD2) and CRD3 of TNFR and that proper folding of PGRN is essential for this binding. Furthermore, dithiothreitol treatment of PGRN, which had been performed by Chen *et al*., abolishes the binding of PGRN to TNFR but enhances its binding to sortilin [16]. Recently, two other groups independently reproduced the binding of PGRN to TNFR1 and TNFR2, and inhibitory effect of this binding on TNF-α-induced effects [17,18]. Dramatic effects of PGRN deficiency have been shown *in vivo* in collagen-induced arthritis and collagen Ab-induced arthritis mouse models, resulting in fulminant courses of disease [14]. Furthermore, the administration of recombinant human PGRN or a recombinant PGRN derivative, antagonist of TNF/TNFR signalling via targeting to TNF receptors (ATSTTRIN), that consists of three modified granulin motifs and their accompanying linker regions [14] had strong anti-inflammatory effects comparable to, or even stronger than, the administration of etanercept [14]. Consequently, PGRN and ATSTTRIN have been regarded as promising next-generation TNF-α blockers [19]. In addition to this strong anti-inflammatory effect mediated by the inhibition of TNFR1 and TNFR2, several other functions of PGRN in humans have been reported [20].

Interestingly, the previously detected PGRN Abs showed neutralizing effects on PGRN plasma levels detected by enzyme-linked immunosorbent assay (ELISA) and Western blot analysis. This observation, given the anti-inflammatory properties of secreted PGRN, suggested a proinflammatory effect of PGRN Abs, which was supported by our observation that the presence of PGRN Abs is associated with active disease state in granulomatosis with polyangiitis [8]. Apart from primary systemic vasculitis, we also found neutralizing PGRN Abs in systemic lupus erythematosus as well as in rheumatoid arthritis [8]. Some of the rheumatoid arthritis patients with PGRN Abs were actually seronegative for RF or anticitrullinated protein Abs. Furthermore, PGRN Abs were detected in patients with spondyloarthritis. This observation led us to conduct the present study to investigate the presence of PGRN Abs in patients with the seronegative disorder PsA, compare it to patients with PsC and healthy controls and investigate possible functional effects of PGRN Abs *in vitro*.

## Methods

### Study participants

This study was approved by our regional ethical review committee (Ethikkommission der Ärztekammer des Saarlandes) and conducted according to the Declaration of Helsinki. Serum samples of patients with PsA were collected prospectively from patients attending three centres of rheumatology between October 2011 and July 2012: Saarland Rheumatology Centre, the Department of Internal Medicine I at University Hospital in Homburg/ 149 Saar, the Rheumatology Department of the University Hospital Frankfurt am Main and the Outpatient Center for Rheumatology in Berlin-Lichtenberg. Sera from patients with PsC were provided by the Department of Dermatology of Saarland University Medical School. Serum samples taken from healthy controls were also obtained at Saarland University Medical School. All serum specimens were stored at −80°C at the Department of Internal Medicine I, José Carreras Research Centre, Saarland University Medical Centre. All patients were examined by a rheumatologist (PsA patients) and a dermatologist (PsC patients) to confirm the diagnosis of PsA according to the CASPAR criteria or to exclude PsA in PsC patients. All diagnoses of PsC were made by dermatologists and confirmed by a rheumatologist. All PsA patients were stratified into subgroups according to gender, age, presence or absence of manifestations of axial disease, enthesitis, dactylitis and therapeutic regimens such as TNF-α blocker–containing medication. Axial disease was defined by positive findings on X-rays or magnetic resonance imaging scans for spondyloarthritis and/or sacroiliitis. Patients were considered positive for enthesitis or dactylitis on the basis of a positive diagnosis during the course of disease; however, no imaging findings have been required. No subgroup stratification for patients with PsC was performed, because the PGRN Ab serostatus of all patients with PsC was negative. All patients and healthy controls gave their written informed consent to participate in the study.

### Progranulin antibody enzyme-linked immunosorbent assay

The ELISA for PGRN Abs was performed as previously described [8]. In short, the *GRN* gene encoding PGRN was recombinantly expressed with a C-terminal FLAG-tag in HEK293 cells under the control of a cytomegalovirus promoter (pSFI). Total cell extracts were prepared and bound to Nunc MaxiSorp plates (eBioscience, Frankfurt, Germany) precoated with murine anti-FLAG mAb at a dilution of 1:2,500 (v/v; Sigma-Aldrich, Munich, Germany) at 4°C overnight. Blocking was performed with 1.5% (w/v) gelatin in Tris-buffered saline (TBS), and washing steps were performed with TBS with Triton X-100. The individual serum samples were diluted 1:100. ELISA was performed according to standard protocols with the following Abs: biotinylated goat antihuman heavy and light chain immunoglobulin G (IgG) at a dilution of 1:2,500 (Dianova, Hamburg, Germany); subclass-specific sheep antihuman IgG1, IgG2, IgG3 and IgG4 (Binding Site Group, Birmingham, UK) at dilutions of 1:5,000; goat antihuman IgM (Dianova) at a dilution of 1:2,500; or goat antihuman IgA (Dianova) at a dilution of 1:2,500. Following this step, corresponding biotinylated secondary Abs were used for immunoassays carried out to detect IgG subclasses and IgM. Peroxidase-labelled streptavidin (Roche Applied Science, Indianapolis, IN, USA) was used at a dilution of 1:50,000. As a cutoff for positivity, the average of the optical density (OD) of the negative samples plus three standard deviations was applied.

### Progranulin plasma levels measured by enzyme-linked immunosorbent assay

PGRN plasma levels were determined with a commercially available ELISA kit (AdipoGen, Incheon, South Korea) according to the manufacturer’s instructions. The median of the plasma PGRN level of the healthy control group was set at 100% [8].

### Cytotoxicity assay

A nonradioactive cytotoxicity assay (EZ4U Cell Proliferation Assay; Biomedica, Vienna, Austria) was performed according to the manufacturer’s instructions. For this TNF-α-induced cytotoxicity assay, we used the highly TNF-α-sensitive mouse fibrosarcoma WEHI-S cell line as the target cells [21]. The interaction of human recombinant PGRN with murine TNFR1 and TNFR2 was previously demonstrated *in vitro* and *in vivo*[14], and, moreover, human PGRN Abs bind murine PGRN (Additional file 1). As a human control target cell line, the TNF-α-sensitive HT-1080 fibrosarcoma cell line was used.

In short, 4 × 10^4^ WEHI-S cells or 4 × 10^4^ HT-1080 cells were seeded into 200 μl of cell culture at 37°C and 5% CO_2_. To detect possible differences between added sera of patients with PsA with and without PGRN Abs, of patients with PsC, and of healthy controls, serum of a PGRN Ab-positive patient with PsA, serum of a matched PGRN Ab-negative patient with PsA, serum of a patient with PsC and serum of a healthy control were added in dilutions from 1:4 to 1:512 to cultured WEHI-S cells and HT-1080 cells, followed by administration of TNF-α (100 pg/ml). Serum samples from gender-, age-, disease- and therapy modality-matched patients were chosen. Serum samples from patients receiving TNF-α blockers or other biologicals were excluded. WEHI-S cells and HT-1080 cells without addition of TNF-α and serum, or solely with addition of TNF-α (100 pg/ml), were used as positive and negative controls. After 24-hour incubation at 37°C, 20 μl of chromophore substrate were added to each well. This chromophore substrate is converted only by vital cells. The adsorption of the product was measured at an OD of 450 nm.

### Statistical analyses

Differences in age and gender between the populations of PsA patients, PsC patients and healthy controls were tested by Student’s *t*-test. Different frequencies of PGRN Abs in the serum samples of patients with PsA and PsC were analysed by χ^2^ test. Differences in the frequency of PGRN Abs between PsA patients after stratification into subgroups were also tested with the χ^2^ test. For analysis of the differences in PGRN plasma levels between healthy controls, PGRN Ab-positive and PGRN Ab-negative patients with PsA and seronegative patients with PsC, the Mann–Whitney *U* test was applied. A two-sided test value less than 0.05 was considered statistically significant. All statistical analyses were performed in SPSS version 19.0 for Windows software (IBM SPSS, Chicago, IL, USA).

## Results

### Patient characteristics

The patients’ characteristics are outlined in Table 1.

**Table 1:** Characteristics of patients with psoriatic arthritis and psoriasis without arthritis

**Characteristics**	**PsA**	**PsC**
Median age (min–max)	54 (24–81)	53 (16–80)
Gender, M/F (%)	43.5/56.4	64/36
Age at psoriasis diagnosis, years	35	33
Age at PsA diagnosis, years	46	
Enthesitis (%)^a^	39.5	
Dactylitis (%)	39.2	
Axial disease (%)	27.7	
Erosive (%)^b^	44.9	
HLA-B27-positive (%)	32.7	
DMARD (%)^c^	48.8	
TNF-α blockers (%)^d^	18.4	

DMARD: disease-modifying antirheumatic drug; HLA-B27: *human leucocyte antigen B27; PsA: psoriatic arthritis; PsC:* psoriasis without arthritic manifestations*; TNF-*α: tumour necrosis factor α. Missing data: ^a^*n* = 69, ^b^*n* = 24, ^c^*n* = 29, ^d^*n* = 9.

### Frequency, titres and immunoglobulin G subclass of progranulin antibodies

**Figure 1:**
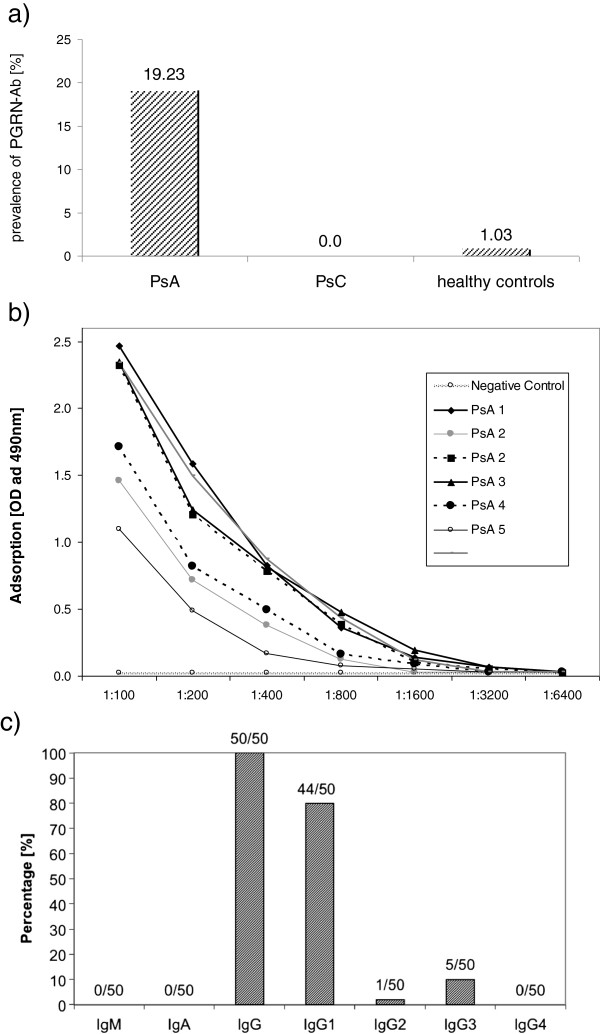
**Occurrence of progranulin antibodies in psoriatic arthritis patients. (a)** Prevalence of progranulin antibodies (PGRN Abs) in psoriatic arthritis (PsA) patients. **(b)** Titres of PGRN Abs in PsA patients. **(c)** Frequency of different immunoglobulin classes of PGRN Abs in PsA patients. Ig: immunoglobulin; OD, optical density; PsC: psoriasis without arthritic manifestations.

Of 260 patients with PsA, 50 (19.23%) had PGRN Abs in their sera. No PGRN Abs were found in the sera of 100 patients with PsC (0%; *P* = 0.0001). Of 97 healthy controls, 1 (1.03%) had PGRN Abs (Figure 1a). PGRN Ab-positive patients had titres ranging from 1:400 to 1:1,600 (Figure 1b). The sera of the 50 PGRN Ab-positive PsA patients were tested for their Ig class. PGRN Abs belonged exclusively to IgG (50 of 50 patients). PGRN Abs were not detected for either IgA or IgM (Additional file 1). Subsequently, IgG subclasses of PGRN Abs were determined. The PGRN Abs in 44 patients (88%) belonged to the IgG1 subclass, 1 (2%) of 50 belonged to the IgG2 subclass, 5 (10%) 50 belonged to the IgG3 subclass and 0 (0.0%) of 50 belonged to the IgG4 subclass (Figure 1c).

### Analysis of the progranulin-positive patients with psoriatic arthritis

**Figure 2:**
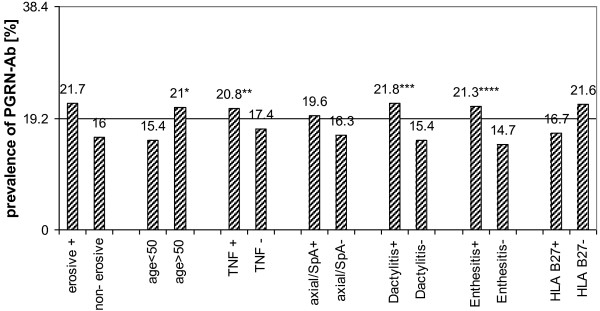
**Progranulin antibodies in subpopulations of patients with psoriatic arthritis.** The columns represent the percentage of progranulin antibody (PGRN Ab)-positive patients in different subgroups of psoriatic arthritis (PsA) compared to the average frequency of PGRN Abs in PsA patients (19.23%; horizontal line). HLA-B27: human leucocyte antigen B27; SpA: spondyloarthritis; TNF: tumour necrosis factor. *P = 0.016, **P = 0.001, ***P = 0.026, ****P = 0.001.

All 260 PsA patients (210 PGRN Ab-negative, 50 PGRN Ab-positive) were stratified according to their clinical manifestations, such as enthesitis, dactylitis, axial manifestations and erosive-proliferative joint damage. Patients with PsA were also analysed in subgroups according to age at primary diagnosis, gender, human leucocyte antigen B27 status and treatment with TNF-α-blocking agents (Figure 2). Although there was no difference between PGRN Ab-positive and PGRN Ab-negative patients with respect to age at the time of PsA diagnosis, PGRN Ab-positive patients were significantly older (>50 years) than PGRN Ab-negative patients with PsA (21% vs. 15.4%; *P* = 0.016). PsA patients with either enthesitis or dactylitis had significantly higher frequencies of PGRN Abs (*P* = 0.001 and *P* = 0.026, respectively, compared to patients with PsA without enthesitis or dactylitis), whereas no statistically significant associations were found for axial manifestations or erosive joint destruction and PGRN Ab positivity. In addition, significantly more patients receiving TNF-α blocker treatment had PGRN Abs compared to patients receiving therapy without TNF-α blockers (20.8% vs. 17.4%; *P* = 0.016).

### Progranulin plasma levels

**Figure 3:**
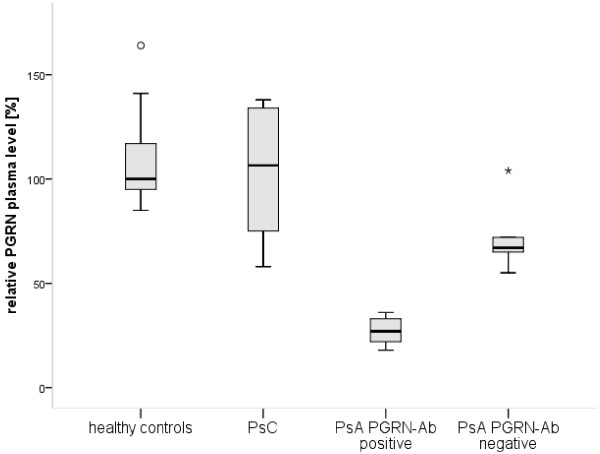
**Association of progranulin antibody status and progranulin plasma levels in psoriasis patients.** Progranulin (PGRN) plasma levels were determined in patients with psoriasis without arthritic manifestations (PsC) (*n* = 10), PGRN antibody (PGRN Ab)-positive patients with psoriatic arthritis (PsA) (*n* = 10), PGRN Ab-negative patients with PsA (*n* = 5) and in healthy controls (*n* = 10). Data are presented as 25% (box), 50% (median) and 75% (box) percentiles and the range (line). Median PGRN plasma level obtained from healthy controls was set at 100%.

PGRN plasma levels were significantly lower in PGRN Ab-positive patients with PsA (*n* = 10) compared to healthy controls (*n* = 10) (*P* < 0.001 by Mann-Whitney *U* test), patients with PsC (*n* = 10) (*P* < 0.001 by Mann-Whitney *U* test) and PGRN Ab-negative patients with PsA (*n* = 5) (*P* < 0.001 by Mann-Whitney *U* test). Furthermore, PGRN Ab-negative patients with PsA had significantly lower plasma levels of PGRN than healthy controls (*P* = 0.019 by Mann-Whitney *U* test). There was also a tendency toward lower PGRN plasma levels in PGRN Ab-negative patients with PsA than in patients with PsC (*P* = 0.055 by Mann-Whitney *U* test) (Figure 3).

### Cytotoxicity assay

**Figure 4:**
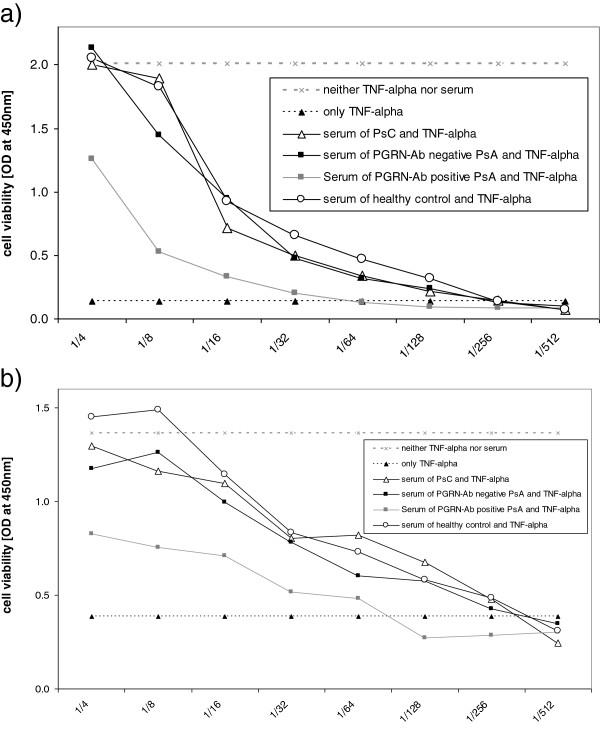
**Tumour necrosis factor α–mediated cytotoxicity dependent on progranulin antibody status. (a)** After administration of serum and TNF-α to WEHI-S cells, the adsorption of coloured formazan, which is a marker for cell viability, was detected at OD of 450 nm. **(b)** After administration of serum and TNF-α to HT-1080 cells, the adsorption of coloured formazan was detected at OD of 450 nm. Ab: antibody; PGRN: progranulin; PsA: psoriatic arthritis; PsC: psoriasis without arthritic manifestations.

In the TNF-α-induced cytotoxicity assays, we analysed the protective effects of sera from healthy controls, patients with PsC and PGRN Ab-positive or PGRN Ab-negative patients with PsA. The addition of sera from healthy controls, patients with PsC and PGRN Ab-negative patients with PsA reduced TNF-α-induced cytotoxicity of WEHI-S and HT-1080 cells to a significantly higher degree than in the sera of PGRN Ab-positive patients with PsA. This difference was significant up to a serum dilution of 1:64 (Figures 4a and 4b).

## Discussion

In the present study, we report the presence of PGRN Abs in relevant titres in a subgroup of patients with PsA (Figures 1a and b). These PGRN Abs had previously been found frequently in primary vasculitis, systemic lupus erythematosus and rheumatoid arthritis, but not at all or very infrequently in controls. Despite the obvious lack of specificity for PsA, PGRN Abs are of particular interest because they had a neutralizing effect on PGRN plasma levels detected by ELISA and Western blot analysis [8]. PGRN is known to be a strong secreted anti-inflammatory mediator [10] by direct inhibition of TNFR1 and TNFR2 [14]. Our results support the hypothesis of a proinflammatory effect of PGRN Abs, as demonstrated by the loss of protective effects of PGRN in the presence of PGRN Abs containing PsA sera in TNF-α-mediated cytotoxicity assays (Figures 4a and 4b). Our results support the observation by Tang *et al*. of a direct inhibitory effect of PGRN on TNFR1 and TNFR2 and that administration of recombinant human PGRN protects cells from cytotoxic effects of TNF-α *in vitro*[14,16]. Given the fact that the half-life of PGRN is about 40 hours [14], in contrast to the short half-life of TNF-α (typical for cytokines) of 20 minutes [22], the effects of the intrinsic TNF-α in the serum of patients and controls is negligible. Our cytotoxicity assays revealed clear differences between the effects of serum from PGRN Ab-positive patients with PsA on the one hand and serum from matched PGRN Ab-negative patients with PsA, patients with PsC or healthy controls on the other hand. The administration of serum samples of patients with PsA with neutralizing PGRN Abs, and thus with lower PGRN levels, protected the WEHI-S and HT-1080 cells from the cytotoxic effects of TNF-α far less than serum samples from patients with PsA without PGRN Abs, patients with PsC or healthy controls. This finding clearly proves the proinflammatory effect of neutralizing PGRN Abs *in vitro*.

The second important finding of the present study is that PGRN Abs were observed in patients with PsA, but not in patients with PsC (Figure 1). Interestingly, Veale *et al*. reported small but significant numbers of B cells in the skin of patients with PsA, but not in the skin of patients with PsC or in healthy controls [7]. In the present study, the occurrence of PGRN Abs in patients with PsA was associated with different clinical characteristics. PsA patients with dactylitis and enthesitis had PGRN Ab more frequently than PsA patients without such manifestations. Moreover, patients with PsA who received TNF-α blocker therapy for at least 3 months had PGRN Abs slightly more frequently (20.8% versus 17.4%), indirectly suggesting that PGRN Abs might be associated with a more aggressive course of disease, necessitating more intensive treatment. Generally, the grades of dactylitis and enthesitis in PsA patients have been suggested to be partly influenced, that is, enhanced, by TNF-α, which is supported by the efficacy of TNF-α blockers in PsA therapy [23-26]. Given the neutralizing effect of PGRN Abs on PGRN plasma levels in PsA patients (Figure 3), as well as in other autoimmune diseases [8], and, more important, given the results of the functional *in vitro* assays indicating a sensitizing effect of PGRN Abs for the effects of TNF-α in patients with PsA, a higher prevalence of PGRN Abs in patients with TNF-α-induced disease manifestations such as enthesitis and dactylitis could obviously be expected. Despite the statistical significance of our results, however, the relative differences in the frequency of PGRN Abs between the various subgroups were rather small (PGRN Ab-positive enthesitis 21.3% vs. PGRN Ab-negative enthesitis 14.7% and PGRN Ab-positive dactilytis 21.8% vs. PGRN Ab-negative enthesitis 15.4%). These results could be explained by the relatively small absolute numbers of patients with subentities and partly by missing data concerning dactylitis and enthesitis. Moreover, we observed a statistically nonsignificant trend between the occurrence of PGRN Abs and the presence of erosive joint disease (*P* = 0.061). In consideration of the suspected pathogenic proinflammatory effect of PGRN Abs disrupting the physiologic homeostasis of TNF-α/PGRN agonists and antagonists in a subgroup of patients with PsA, PGRN Abs might be of use as prognostic markers for the course of disease and/or as predictive markers for the effectiveness of TNF-α-blocking agents. Theoretically, the identification of neutralizing PGRN Abs in PsA could eventually lead to a more individualized therapy because patients with PGRN Abs have lower physiologic TNF-α antagonist levels and might profit from dose intensification of TNF-α blockers. From this point of view, prospective studies of patients with PsA are needed to evaluate PGRN Abs as possible biomarkers for the diagnosis, risk stratification and choice of adequate treatment modality.

## Conclusion

Neutralizing PGRN Abs occurred in relevant titres in a subgroup of patients with PsA, but not in PsC patients. PGRN Ab-positive patients with PsA had more frequently enthesitis and dactylitis than PGRN Ab-negative patients with PsA. Moreover in TNF-α-induced cytotoxicity assays using WEHI-S and HT-1080 cells, the protective effects of PGRN were inhibited by PGRN Ab-containing sera of patients with PsA. This suggests that PGRN Abs not only may be useful as a diagnostic and prognostic marker but also may be involved in the pathogenesis of the arthritic process in a subgroup of patients with PsA.

## Abbreviations

Ab: Antibody; IgG: Immunoglobulin G; PGRN: Progranulin; PsA: Psoriatic arthritis; PsC: Psoriasis without arthritic manifestations; TNF-α: Tumour necrosis factor α; TNFR: Tumour necrosis factor receptor.

## Competing interests

Saarland Universities Patent Marketing Agency, LT, KDP and MP filed 61/730,772, which covers means and methods for detecting autoimmune disorders in which progranulin may be involved.

## Authors’ contributions

MZ, NF and ER performed most of the experiments and were involved in data analysis. LT, MP and GA were involved in the study design and data analysis and drafted the manuscript. MFO was involved in data analysis. All authors read and approved the final manuscript.

## Supplementary Material

Additional file 1: Figure S1 Immunoglobulin class of progranulin (PRGN) antibodies (Abs) in psoriatic arthritis. Each PGRN Ab-containing serum was tested for immunoglobulin (Ig) class of PGRN Abs. **(a)** PGRN Abs were tested for IgG class. **(b)** PGRN Abs were tested for IgA class. **(c)** PGRN Abs were tested for IgM class. **(d)** PGRN Abs were tested for IgG1 subclass. **(e)** PGRN Abs were tested for IgG2 subclass. **(f)** PGRN Abs were tested for IgG3 subclass. **(g)** PGRN Abs were tested for IgG4 subclass. Sera were used at a dilution of 1:100. Click here for file
