# Supplementary material for: Progranulin antibodies entertain a proinflammatory environment in a subgroup of patients with psoriatic arthritis
Source: Arthritis Res Ther. 2013 Dec 10;15(6):R211. doi: 10.1186/ar4406 (PMC3978758; doi:10.1186/ar4406)
Supplement: Additional file 1: Figure S1 — Immunoglobulin class of progranulin (PRGN) antibodies (Abs) in psoriatic arthritis. Each PGRN Ab-containing serum was tested for immunoglobulin (Ig) class of PGRN Abs. (a) PGRN Abs were tested for IgG class. (b) PGRN Abs were tested for IgA class. (c) PGRN Abs were tested for IgM class. (d) PGRN Abs were tested for IgG1 subclass. (e) PGRN Abs were tested for IgG2 subclass. (f) PGRN Abs were tested for IgG3 subclass. (g) PGRN Abs were tested for IgG4 subclass. Sera were used at a dilution of 1:100. [file ar4406-S1.doc]

**Additional file**

Supplementary Figure 1a)

Supplementary Figure 1b)

Supplementary Figure 1c)

Supplementary Figure 1d)

Supplementary Figure 1e)

Supplementary Figure 1f)

Supplementary Figure 1g)

Additional file 1: Figure S1 Immunoglobulin class of Progranulin-antibodies in PsA

Each PGRN-Ab containing serum was tested for Ig-class of PGRN-Abs. a) PGRN-Abs were tested for IgG class. b) PGRN-Abs were tested for IgA class. c) PGRN-Abs were tested for IgM class. d) PGRN-Abs were tested for IgG1 subclass. e) PGRN-Abs were tested for IgG2-subclass. f) PGRN-Abs were tested for IgG3-subclass. g) PGRN-Abs were tested for IgG4-subclass. Sera were used at a dilution of 1:100.
